# Increased Sperm DNA Damage in Experimental Rat Varicocele Model and The Beneficial Effect of Varicocelectomy

**Published:** 2012-06-19

**Authors:** Metin İshak Öztürk, Orhan Koca, Muzaffer Oğuz Keleş, Seda Yılmaz, Muhammet Ihsan Karaman

**Affiliations:** 1 Department of Urology, Haydarpasa Numune Training and Research Hospital, Istanbul, Turkey; 2 Gebze Institute of High Technology Molecular Biology and Genetics, Kocaeli, Turkey

**Keywords:** Varicocele, Sperm, DNA Fragmentation, Sperm Chromatin Dispersion Test

## Abstract

**Background:**

Varicocele, the abnormal dilatation of the veins in the pampiniform plexus is commonly seen in infertile patients. In this study, we aim to examine sperm DNA damage after the creation of experimental varicocele in rats and to observe the change of this damage after a varicocelectomy.

**Materials and Methods:**

In this experimental study, a total of 30 adult male Wistar albino rats were divided into three groups. The 10 rats in group 1 underwent a sham operation, an experimental varicocele was created in both the10 rats in group 2 and the 10 rats in group 3 (a total of 20 rats). While the rats of group 2 were sacrificed after four weeks, the rats in group 3 underwent a varicocelectomy after four weeks and were sacrificed four weeks after the varicocelectomy to observe its effects. Sperm DNA fragmentation was assessed with a Halomax® kit. The DNA Fragmentation Index (DFI) was calculated and the groups were compared according to their DFI. Statistical analysis was performed using the Mann-Whitney U test.

**Results:**

Median sperm DFI was 17.6 (range: 7.6) in the right testicle and 18.3 (range: 6.8) in the left testicle in the control group; 30.7 (range: 8.8) in the right testicle and 31.8 (range: 9.6) in the left testicle in the varicocele group; 27.1 (range: 8.1) in the right testicle and 28.6 (range: 8.9) in the left testicle in the varicocelectomy group. DNA damage in both right and left testicles was statistically significant between the three groups (p<0.05).

**Conclusion:**

The results of this study show that varicocele leads to increased sperm DNA damage and this damage is decreased by varicocelectomy.

## Introduction

Varicocele is the abnormal dilatation of the veins in the pampiniform plexus. While its incidence is reported to be 4.4-22.6% in the general population, an incidence of 21-41% in infertile patients and 75-81% in secondary infertile patients has been reported (1). Although varicocele has been described for more than one hundred years, the mechanisms by which varicocele causes infertility are still unclear (2). The effect of varicocele on fertility probably results from a multifactorial interaction in which more than one mechanism are involved (3).

Several theories have been suggested to explain the relationship between varicocele and infertility. The theories accepted include: the increase of arterial blood flow and testicular temperature caused by varicocele (4); venous, stasis, renal, and adrenal toxic metabolites (5); the decrease of intratesticular and/or peripheral testosterone level caused by varicocele (6); and increased DNA damage observed in patients with varicocele (7).

In the last phase of spermatogenesis, spermatid nuclear remodeling and condensation is associated with the displacement of nuclear histones by transition proteins and then by protamines. Protamines provide sperm head condensation and DNA stabilization and allow for denser packaging of DNA in spermatozoon than histones (8). Impaired spermatogenesis may cause the production of spermatozoa with highly damaged DNA (9, 10).

While the incidence of sperm with DNA damage is approximately 10% in fertile males, the corresponding rate is about 20-30% in infertile males (11, 12). Although there are studies reporting that sperm DNA damage has no effect on pregnancy rates, some studies report that high sperm DNA damage leads to a decrease in pregnancy rates, including spontaneous pregnancy and pregnancy after assisted reproduction (13-15). Information obtained from humans about this issue is limited due to ethical reasons, such as the inability to perform testicular biopsy on every patient. In this study, we aim to examine sperm DNA damage after the creation of experimental varicocele in rats and to observe the change of this damage after varicocelectomy.

## Materials and Methods

In this experimental study, after obtaining approval from the Ethics Committee, a total of 30 adult male Wistar albino rats were randomized into three groups. In group 1, 10 rats underwent a sham operation and were classified as the control group. Experimental varicoceles were created in both the 10 rats in group 2 and the 10 rats in group 3 (a total of 20 rats), using the method described by Saypol et al. (16). When the rats of groups 2 and 3 were being reoperated, the diameter of the spermatic vein was measured. If the spermatic vein diameter was at least 1 mm, the varicocele was considered to be successfully created (17).

After four weeks the rats in group 2 were sacrificed, while the rats in group 3 underwent a varicocelectomy and were sacrificed four weeks later to observe the effects of the varicocelectomy. The total number of rats was maintained by replacing the rats that did not develop a varicocele or those that died before the sacrifice with new ones.

All rats were fed using standard 8 mm pellet feed and maintained in a constant environment with a 12: 12 hour light: dark cycle. All animals were given ad libitum water and feed. Room temperature was 22 ± 2˚C and humidity rate was 50 ± 10%.

### Creation of experimental varicocele model

The surgical procedure was performed according to rules of antisepsis. The body temperature of the animals was monitored with a rectal thermometer; we attempted to maintain the temperature be kept at 37˚C.

The rats were weighed. For general anesthesia, ketamine (10 mg/100 g) and chlorpromazine (1 mg) was administered intraperitoneally.

In the rats that underwent the experimental varicocele, an 0.8 mm-thick metal wire probe was placed along the renal vein. A 4-0 silk suture was ligated around the renal vein and metal wire probe. When the metal wire probe was removed, a nearly 50% narrowing of the renal vein was observed. After tying the ligature the probe was removed, which produced an approximately 50% decrease in renal vein diameter. In the sham group, the rats underwent a similar procedure, but we did not ligate the renal vein.

After four weeks we checked for the occurrence of varicocele. At the end of four weeks, dilated left spermatic veins were seen in 18 of 20 rats that underwent laparotomy. In 10 rats, dilated spermatic vein was ligated using 4/0 silk and cut (varicocelectomy). Tunica albuginea was opened at the fourth week in groups 1 and 2. In the eighth week in group 3, tunica albuginea was opened, testicular tissue was obtained, and the rats were sacrificed by cervical dislocation. The testicular tissue samples obtained were put in hydroxyethyl piperazineethanesulfonic acid (HEPES) buffered medium (G-IVF, Vitrolife, Sweden).

### Sperm DNA fragmentation assessment

Sperm DNA fragmentation was assessed with a Halomax® kit (Halotech DNA, Spain). An aliquot of testicular tissue was diluted to 15-20×106/ml in phosphate-buffered saline (PBS). Agarose (in eppendorf tubes provided with the kit) was placed in a water bath at 95˚C -100˚C for 5 minutes to fuse the agarose, and then in a water bath at 37˚C until the temperature equilibrated. After 5 minutes of incubation at 37˚C, 25 μL of the diluted tissue sample was added to a 50 μl agarose-filled eppendorf tube at 37˚C and mixed with the fused agarose. 1.5-2 μL of tissue-agarose mixture was pipetted onto slides precoated with agarose (provided in the kit) and then covered with a 24 × 24 mm coverslip. The slides were placed on a cold plate in the refrigerator at 4˚C for 5 minutes to allow the agarose to produce a microgel that had the sperm cells embedded. The coverslips were removed and slides were immersed horizontally in a lysis solution (LS) prepared by mixing 7 μL of reducing agent (RA) with 1 mL of base lysis solution (BLS) provided in the kit and incubated for 5 minutes. After washing for 5 minutes with distilled water, the slides were dehydrated in increasing concentrations of ethanol (70-100%) for 2 minutes and then left to dry. Slides were stained with a mixture of Wright’s staining solution (Merck, Germany) and PBS (Merck, Germany) (1:1) for 5-10 minutes, washed with water, and then dried. A total of 100 spermatozoa per sample were analyzed by brightfield microscopy (×100 objective). Sperm with large, spotty halos of chromatin dispersion were classified as ’spermatozoa having DNA fragmentation' and those with small, compact halos of chromatin dispersion were classified as ’spermatozoa having no fragmentation'. DNA Fragmentation Index (DFI) was calculated and the groups were compared for DFI.

### Statistical analysis

All data were analyzed by SPSS software (Version 13.0, SPSS Inc., Chicago, IL, USA). Statistical analysis was performed using the Mann-Whitney U test. P<0.05 was considered statistically significant.

## Results

**Fig 1:**
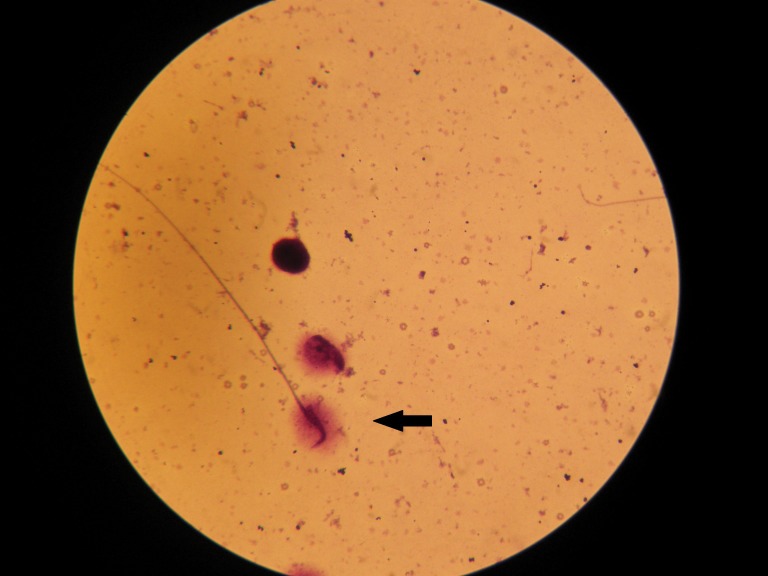
Sperm cells with DNA fragmentation seen with the halo structure surrounding the head.

**Fig 2:**
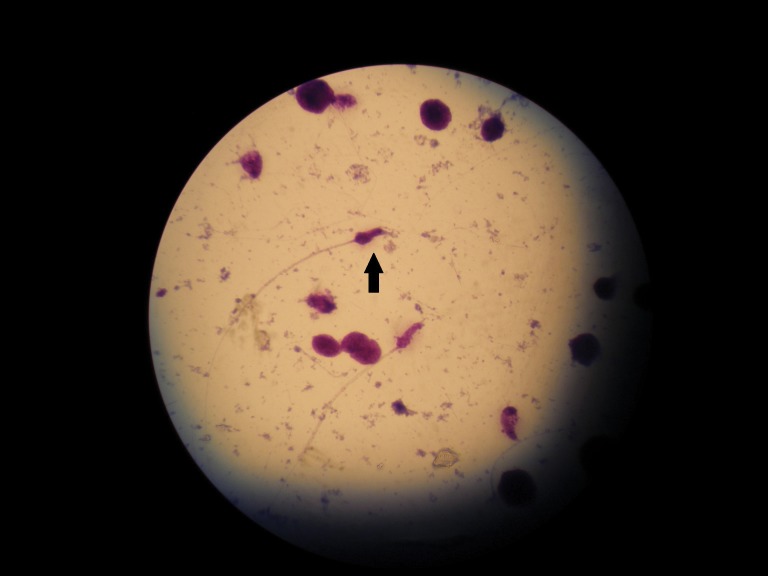
Sperm cells with no DNA fragmentation seen without the halo structure surrounding the head.

After staining the slides with Wright’s solution some of the sperm cells presented a halo structure, which indicated the presence of fragmented DNA; some had no halo (DNA intact; Figs 1, 2).

Median sperm DFI was 17.6 (range: 7.6) in the right testicle, 18.3 (range: 6.8) in the left testicle, and 18.3 (range: 7.9) in both testicles of the control group. No statistically significant difference was found between the right and left testicles in the control group (p>0.05).

Median sperm DFI was 30.7 (range: 8.8) in the right testicle, 31.8 (range: 9.6) in the left testicle, and 31.8 (range: 10.1) in both testicles of the varicocele group. No statistically significant difference was found between the right and left testicles in the varicocele group (p>0.05).

Median sperm DFI was 27.1 (range: 8.1) in the right testicle, 28.6 (range: 8.9) in the left testicle, and 27.1 (range: 9.0) in both testicles of the varicocelectomy group. No statistically significant difference was found between the right and left testicles in the varicocelectomy group (p>0.05).

A statistically significant difference was found between the three groups in terms of sperm DFI in both the right and left testicles (Table 1).

Because there was no significant difference between right and left testicles in the three groups, the differences between the control and varicocele groups (p=0.028), control and varicocelectomy groups (p=0.036), and varicocele and varicocelectomy groups (p=0.048) were statistically significant when the right and left testicles were evaluated together.

**Table 1:** Sperm DNA fragmentation rates


	Control group (n=10)		Varicocele group (n=10)		Varicocelectomy group (n=10)		P value

	Median	Range	Median	Range	Median	Range	

**Right testicle**	17.6	7.6	30.7	8.8	27.1	8.1	<0.05*
**Left testicle**	18.3	6.8	31.8	9.6	28.6	8.9	<0.05*
**Both testicles**	18.3	7.9	31.8	10.1	27.1	9.0	<0.05*
**P value**	>0.5		>0.05		>0.05		


* P values of right (R), left (L), and both (B) testes. Control group-varicocele group: p=0.026 (R), p=0.028 (L), p=0.028 (B); Con-trol group-varicocelectomy group: p=0.032 (R), p=0.032 (L), p=0.036 (B); Varicocele group-varicocelectomy group: p=0.046 (R), p=0.046 (L), p=0.048 (B).

## Discussion

The relationship between varicocele and infertility has not been fully elucidated. All patients with varicocele are not infertile and all patients who undergo varicocelectomy do not become fertile. Although it has been known for many years that varicocele may impair spermatogenesis (18), this information is not enough to explain the association between varicocele and infertility. It has been reported that increased testicular temperature and reactive oxygen species (ROS) levels observed in varicocele might cause increased sperm DNA damage (19).

This study aimed to examine the effects of varicocele and varicocelectomy on sperm DNA damage. While no statistically significant difference was found between the right and left testicles in all three groups, a statistically significant difference was found in the three groups in terms of sperm DFI.

Marmar have suggested that a varicocele could be a secondary lesion that accompanies an underlying genetic disorder, which impairs spermatogenesis and contributes to infertility (3). Considering this hypothesis, in our study we have divided the rats into three groups and compared the effects of varicocele and varicocelectomy on sperm DNA damage with each other and with the group that underwent the sham operation.

In the experimental varicocele model created by Barqawi et al. they evaluated apoptosis at the 7^th^, 14^th^, and 28^th^ day of the study in the testicles of rats and found maximum apoptosis on the 28^th^ day (2). Fazlıoglu et al. reported that in the varicocelectomy model in rats, the apoptotic index was significantly decreased on the 21^st^ day, which approximated to the level of the control group on the 28^th^ day (20). In our study, we waited for a four-week period for the formation of varicocele, and another four-week period for recovery following varicocelectomy. In their experimental varicocele model, Luo et al. have reported that some parameters (apoptosis index of Leydig cells and StAR mRNA levels) became significantly different from the control group in the fourth week and some (apoptosis index of Leydig cells, StAR mRNA levels and intratesticular testosterone levels) in the eighth week, this may suggest that our four week period may have not have been adequate (21).

One of the interesting features of the varicocele is that, even in a unilateral varicocele, both testicles develop damage (3). Gat et al., whose patients with unilateral varicocele underwent physical examinations, reported the incidence of bilateral varicocele as 80% and suggested that bilateral damage might be attributed to this (22). However, in our study, the observation of increased sperm DNA damage in both testicles, despite the formation of a left varicocele alone, suggested that bilateral testicular damage resulted from mechanisms other than the presence of a bilateral varicocele.

The integrity of sperm DNA is important for male fertility potential. Evenson et al. have reported that the probability of spontaneous pregnancy was lower in couples in whom the sperm DNA damage rate was above 30% (23). In addition, it has been demonstrated that the rate of sperm with damaged DNA was higher in couples with pregnancies terminated due to miscarriage compared to fertile couples (24). Ahmadi et al. formed damaged DNA in hamster sperm using radiation, and reported that sperm maintained its potential for fertilization regardless of the DNA damage if the damage rate was below 8%. In this case, DNA repair mechanisms of the ovum were involved and the pregnancy occurred; but if the damage rate exceeded 8%, the pregnancy resulted in a lower embryonic development and a higher early pregnancy loss rate (25).

The sperm chromatin dispersion (SCD) test is a reliable method used to evaluate mammalian sperm DNA fragmentation (26-29). Halosperm is a new improved SCD test developed by Fernandez et al. (30). In our study, we have compared sperm DNA damage using a Halomax kit. DFI was calculated and the groups were compared according to DFI.

Diagnosis and follow up of infertile patients is generally performed using semen analysis. Although semen analysis provides valuable information, it has some limitations. Semen parameters show both biological, intra-, and inter-observer variations (31) and sperm quality is mainly evaluated based on motility and morphology. However, it has been demonstrated that in infertile patients, sperm with normal morphology have high rates of DNA fragmentation (32). DFI provides additional information about the potential of fertility and shows less biological variation when compared to conventional semen analysis (33). Therefore in our study we have compared DFI scores and demonstrated that DNA damage was more prevalent in the group with varicocele compared to the sham group. As a result of varicocelectomy the damage decreased.

Although sperm DNA damage has been known for many years, the acceptable rate of sperm with DNA damage in humans is controversial. Evenson et al. have determined that the likelihood of spontaneous pregnancy was lower in couples with a DFI>30%, reasonable in couples with a DFI between 15-30%, and higher in those with a DFI<15% (23). Spanò et al. have reported that the incidence of spontaneous pregnancy was significantly decreased in the couples with a sperm DFI>40% (14) and Bungum et al. suggested that incidences of pregnancy and delivery following IUI were significantly higher in the couples with a sperm DFI<27% (15).

It has been suggested that sperm DNA damage could be a late outcome of increased ROS levels, and therefore its resolution could take time (7, 34). Zini et al. have shown a statistically insignificant increase in the mean sperm concentration and percent of progressive sperm motility after microsurgical varicocelectomy, and a statistically significant decrease in sperm DNA damage (35). In addition, they have shown an improvement in sperm DNA integrity at the fourth month with continued improvement at the sixth month follow up after varicocelectomy. In our study, sperm DNA damage showed a regression after varicocelectomy, but did not reach the level of the sham group. This may be explained by either the short time interval that had passed after the varicocelectomy or the irreversible nature of the damage caused by the varicocele. This issue can be enlightened with future studies that include a larger number of subjects and a longer waiting period after the varicocelectomy.

The limitations of this study include the small number of rats used due to ethical reasons, observations of sperm DNA damage examined after different time periods following varicocelectomy, and the lack of an evaluation on the effects of sperm DNA damage on pregnancy rates.

## Conclusion

The results of this study demonstrate that varicocele causes increased sperm DNA damage and varicocelectomy decreases this damage. Thus, it should be considered that sperm DNA damage which increased by varicocele may be decreased by varicocelectomy.

## References

[1] Will MA, Swain J, Fode M, Sonksen J, Christman GM, Ohl D (2011). The great debate: varicocele treatment and impact on fertility. Fertil Steril.

[2] Barqawi A, Caruso A, Meacham RB (2004). Experimental varicocele induces testicular germ cell apoptosis in the rat. J Urol.

[3] Marmar JL (2001). The pathophysiology of varicoceles in the light of current molecular and genetic information. Hum Reprod Update.

[4] Hurt GS, Howards SS, Turner TT (1986). Repair of experimental varicoceles in the rat.Long-term effects on testicular blood flow and temperature and cauda epididymidal sperm concentration and motility. J Androl.

[5] Benoff S, Gilbert BR (2001). Varicocele and male infertility: part I.Preface. Hum Reprod Update.

[6] Zheng Y, Zhang X, Zhou J, Cheng F, Zhou B (2008). Effects on the ipsilateral testis during progression of experimental varicocele in rat. Med Sci Monit.

[7] Smit M, Romijn JC, Wildhagen MF, Veldhoven JL, Weber RF, Dohle GR (2010). Decreased sperm DNA fragmentation after surgical varicocelectomy is associated with increased pregnancy rate. J Urol.

[8] Steger K, Pauls K, Klonisch T, Franke FE, Bergmann M (2000). Expression of protamine-1 and -2 mRNA during human spermiogenesis. Mol Hum Reprod.

[9] Zini A, Blumenfeld A, Libman J, Willis J (2005). Beneficial effect of microsurgical varicocelectomy on human sperm DNA integrity. Hum Reprod.

[10] Tavalaee M, Nasr-Esfahani MH, Deemeh MR (2008). Etiology and Evaluation of Sperm Chromatin Anomalies. Int J Fertil Steril.

[11] Zini A, Bielecki R, Phang D, Zenzes MT (2001). Correlations between two markers of sperm DNA integrity, DNA denaturation and DNA fragmentation, in fertile and infertile men. Fertil Steril.

[12] Zini A, Fischer MA, Sharir S, Shayegan B, Phang D, Jarvi K (2002). Prevalence of abnormal sperm DNA denaturation in fertile and infertile men. Urology.

[13] Gandini L, Lombardo F, Paoli D, Caruso F, Eleuteri P, Leter G (2004). Full-term pregnancies achieved with ICSI despite high levels of sperm chromatin damage. Hum Reprod.

[14] Spanò M, Bonde JP, Hjøllund HI, Kolstad HA, Cordelli E, Leter G (2000). Sperm chromatin damage impairs human fertility.The Danish First Pregnancy Planner Study Team. Fertil Steril.

[15] Bungum M, Humaidan P, Spano M, Jepson K, Bungum L, Giwercman A (2004). The predictive value of sperm chromatin structure assay (SCSA) parameters for the outcome of intrauterine insemination, IVF and ICSI. Hum Reprod.

[16] Saypol DC, Howards SS, Turner TT, Miller ED Jr (1981). Influence of surgically induced varicocele on testicular blood flow, temperature, and histology in adult rats and dogs. J Clin Invest.

[17] Lee JD, Lee TH, Cheng WH, Jeng SY (2009). Involved intrinsic apoptotic pathway of testicular tissues in varicocele-induced rats. World J Urol.

[18] Gorelick JI, Goldstein M (1993). Loss of fertility in men with varicocele. Fertil Steril.

[19] Saleh RA, Agarwal A, Sharma RK, Said TM, Sikka SC, Thomas AJ Jr (2003). Evaluation of nuclear DNA damage in spermatozoa from infertile men with varicocele. Fertil Steril.

[20] Fazlioglu A, Yilmaz I, Mete O, Kurtulus F, Parlakkilic O, Güctas O (2008). The effect of varicocele repair on experimental varicocele-induced testicular germ cell apoptosis. J Androl.

[21] Luo DY, Yang G, Liu JJ, Yang YR, Dong Q (2011). Effects of varicocele on testosterone, apoptosis and expression of StAR mRNA in rat Leydig cells. Asian J Androl.

[22] Gat Y, Bachar GN, Zukerman Z, Belenky A, Gornish M (2004). Varicocele: a bilateral disease. Fertil Steril.

[23] Evenson DP, Jost LK, Marshall D, Zinaman MJ, Clegg E, Purvis K (1999). Utility of the sperm chromatin structure assay as a diagnostic and prognostic tool in the human fertility clinic. Hum Reprod.

[24] Carrell DT, Liu L, Peterson CM, Jones KP, Hatasaka HH, Erickson L (2003). Sperm DNA fragmentation is increased in couples with unexplained recurrent pregnancy loss. Arch Androl.

[25] Ahmadi A, Ng SC (1999). Fertilizing ability of DNA-damaged spermatozoa. J Exp Zool.

[26] Velez de la Calle JF, Muller A, Walschaerts M, Clavere JL, Jimenez C, Wittemer C (2008). Sperm deoxyribonucleic acid fragmentation as assessed by the sperm chromatin dispersion test in assisted reproductive technology programs: results of a large prospective multicenter study. Fertil Steril.

[27] de la Torre J, López-Fernández C, Pita M, Fernández JL, Johnston SD, Gosálvez J (2007). Simultaneous observation of DNA fragmentation and protein loss in the boar spermatozoon following application of the sperm chromatin dispersion (SCD) test. J Androl.

[28] Johnston SD, López-Fernández C, Gosálbez A, Zee Y, Holt WV, Allen C (2007). The relationship between sperm morphology and chromatin integrity in the koala (Phascolarctos cinereus) as assessed by the Sperm Chromatin Dispersion test (SCDt). J Androl.

[29] Deemeh MR, Tavalee M, Razavi S, Nasr-Esfahani MH (2007). Evaluation of Protamine Deficiency and DNA Fragmentation in Two Globozoospermia Patients Undergoing ICSI. Int J Fertil Steril.

[30] Fernández JL, Muriel L, Goyanes V, Segrelles E, Gosálvez J, Enciso M (2005). Simple determination of human sperm DNA fragmentation with an improved sperm chromatin dispersion test. Fertil Steril.

[31] Alvarez C, Castilla JA, Martínez L, Ramírez JP, Vergara F, Gaforio JJ (2003). Biological variation of seminal parameters in healthy subjects. Hum Reprod.

[32] Avendaño C, Franchi A, Taylor S, Morshedi M, Bocca S, Oehninger S (2009). Fragmentation of DNA in morphologically normal human spermatozoa. Fertil Steril.

[33] Evenson DP, Larson KL, Jost LK (2002). Sperm chromatin structure assay: its clinical use for detecting sperm DNA fragmentation in male infertility and comparisons with other techniques. J Androl.

[34] Dada R, Shamsi MB, Venkatesh S, Gupta NP, Kumar R (2010). Attenuation of oxidative stress & DNA damage in varicocelectomy: implications in infertility management. Indian J Med Res.

[35] Zini A, Azhar R, Baazeem A, Gabriel MS (2011). Effect of microsurgical varicocelectomy on human sperm chromatin and DNA integrity: a prospective trial. Int J Androl.

